# Hybrid HPC and Quantum Computing Framework for Multiscale Modeling of Chemical Systems

**DOI:** 10.1021/acs.jctc.6c00542

**Published:** 2026-08-05

**Authors:** Joseph C. R. Thacker, Mateusz Meller, Alberto Popescu, Andrew Patterson, Róbert Izsák, Vendel Szeremi, Thomas W. Keal

**Affiliations:** † STFC Scientific Computing, 97005 Daresbury Laboratory, Keckwick Lane, Daresbury, Warrington WA4 4AD, U.K.; ‡ STFC Hartree Centre, Daresbury Laboratory, Keckwick Lane, Daresbury, Warrington WA4 4AD, U.K.; § Riverlane Ltd., Cambridge CB2 3BZ, U.K.

## Abstract

We present a multiscale workflow for coupling quantum computing algorithms for quantum chemical calculations with conventional high-performance computing (HPC) software. The resulting embedding framework, implemented in the multiscale computational chemistry environment ChemShell, combines a generic *N*-layer subtractive quantum mechanics/molecular mechanics (QM/MM) scheme and selection of an active orbital space for the quantum algorithm region. We have benchmarked the embedding models for gas phase and solvated molecules to understand the performance, benefits and current limitations of embedding quantum computing algorithms within a QM/MM model. We compare the performance of simulated quantum phase estimation (QPE) calculations on an HPC cluster using graphical processing units (GPUs) and central processing units (CPUs).

## Graphical abstract

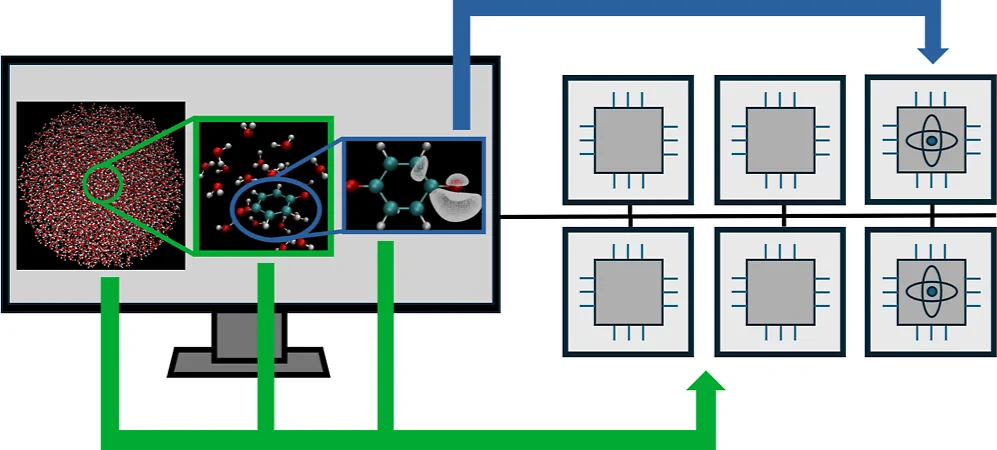

## Introduction

Solving the electronic structure problem and its derivatives is of central interest to computational chemistry as in principle this allows all properties of a chemical system to be calculated. However, solving the many-body Schrödinger equation analytically is not possible except in a few simple cases, and numerical solutions to the problem rapidly become prohibitive on conventional hardware as the system size increases due to exponential time complexity scaling. To mitigate this, many powerful methods have been devised to find approximate solutions within practical timeframes and polynomial time complexities.^1–6^ Nevertheless, there is a strong motivation to further develop techniques that are both highly accurate and can tackle large systems. Employing quantum computers (and associated quantum algorithms) is one possible candidate. Compared to conventional devices, quantum computers operate on quantum bits (qubits), which can be in a superposition and entangled. Additionally, the amplitudes of the resulting wave function can interfere. This in turn, could enable quantum advantage, that is, algorithmic scaling advantage coming from exploitation of quantum characteristics.^7^

In recent years there has been a lot of focus on finding practical quantum advantage (i.e., quantum utility) in varied problems across science.^8^ Early potential candidates include problems in chemistry, material science, drug discovery, combinatorial optimization, and machine learning.^9–12^ However, even though there has been steady progress in the development of quantum computing hardware, we are still in the era of noisy intermediate scale quantum (NISQ) devices.^13^ NISQ devices are heavily influenced by noise, have short coherence time, and thus lack capability for error-correcting codes, limiting the number of useful algorithms that can be run. Nevertheless, in the field of quantum computational chemistry (that is, quantum chemical calculations performed on quantum computers) there are a number of practical algorithms and implementations to solve the electronic structure for small molecules.^14–16^ For extensive reviews of the methods and progress in this field, the reader is referred to refs 9 and 10 .

In the context of quantum chemistry, current quantum computing applications predominantly focus on variational quantum eigensolvers (VQE),^14^ due to their applicability to NISQ devices. Yet, from the computational chemistry perspective, it is important to develop software frameworks that can take advantage both of emergent algorithms and emergent quantum hardware. Indeed, supporting novel accelerators (even established ones like GPUs) is highly nontrivial and it is essential that workflows that integrate quantum devices are developed in parallel and if possible anticipate hardware developments. Therefore, existing and prospective frameworks, should also allow using quantum algorithms implementable on fault-tolerant hardware of the future. One example of such an algorithm is quantum phase estimation (QPE).^17^ In this work, we present a workflow for hybrid HPC–quantum computing calculations, which we demonstrate using the QPE algorithm based on work by Somma,^18^ implemented in the Discover Lite package. Moreover, insights into resource requirements of fault-tolerant quantum algorithms can guide codesign of novel programming models for hybrid HPC–quantum computing systems.

To address the challenges associated with scaling quantum computing algorithms to larger chemical systems, we propose here to embed quantum computing calculations in conventional computational chemistry calculations on HPC, using multiscale modeling approaches. Multiscale chemistry methods, typically combining quantum mechanics with classical molecular mechanics (QM/MM), are widely used to address the poor scaling/performance of quantum chemical calculations (e.g., density functional theory and wave function methods) for large molecules. To make use of QM/MM^19–21^ methods, one requires a problem where highly accurate computation is only required for a small subset of atoms in the system, where a localized chemical process occurs. Problems such as the reactivity of molecules in solution,^22^ protein–ligand binding^23,24^ and material defects^25^ are all highly amenable to multiscale approaches.

In this paper we first describe the QPE method, after which we describe the multiscale embedding approaches used in our framework, the atomistic-subtractive scheme and the selection of an active orbital space for QPE calculations, and how these may be combined for maximum flexibility in a 4-layer embedding scheme. We then give an overview of the workflow as implemented in the multiscale computational chemistry environment ChemShell.^26,27^ After describing the methods, we present benchmarks for the embedding schemes developed. The first benchmark is a series of small molecules with no embedding; the second benchmark is a 3-layer embedding (QPE/DFT/MM) of a water molecule within a larger spherical droplet of water; and the third benchmark is a 4-layer embedding scheme (QPE/CCSD/DFT/MM), where the two innermost layers are embedded using orbital based methods, which are then embedded atomistically within the final two layers.

## Methods

### Electronic Structure Problem

Electronic structure calculations aim to find energy eigenstates of a given Hamiltonian, and corresponding eigenvalues describing energy levels of the molecular system.^10,28,29^ By employing the Born–Oppenheimer approximation, and treating the atomic nuclei as point charges, we can write the electronic Schrödinger equation as follows

1 $${Ĥ}_{elec}\Psi =E_{elec}\Psi$$

where the electronic Hamiltonian is defined as

2 $${Ĥ}_{elec}=-\sum_{i=1}^{N}\left(\frac{1}{2}\nabla_{i}^{2}-\sum_{A=1}^{M}\frac{Z_{A}}{r_{iA}}+\sum_{j=1}^{j<i}\frac{1}{r_{ij}}\right)$$

By solving the Schrödinger equation for the system at hand, an expectation value can be calculated which corresponds to the ground state energy.

Unfortunately, solving the Schrödinger equation analytically is often impossible, and the exact simulation is computationally hard, with exponentially scaling complexity in terms of time and resources.

Due to the Pauli exclusion principle, the wave function must be antisymmetric under the exchange of two electrons.^10,28,29^ A natural way to achieve this is via second quantization, which preserves the exchange statistics based on the operators that are applied to the wave function.^10,28,29^ Starting from projecting the Hamiltonian onto the basis of wave functions, we can use a simplified version of Slater determinants to describe which spin–orbitals are occupied by the electrons.^10,28^ Then we define two Fermionic operators: a creation operator *a*_*p*_^†^ which creates an electron within spin–orbital *p*, and an annihilation operator *a*_*p*_, which removes an electron from spin–orbital *p*. The creation and annihilation operators obey Fermionic anticommutation relations.

As the number of electrons must be conserved, the second quantized operators must contain an equal number of Fermionic annihilation and creation operators.^28,29^ We can define electronic Hamiltonian in the second quantized form as

3 $${Ĥ}_{elec}=\sum_{pq}h_{pq}a_{p}^{†}a_{q}+\frac{1}{2}\sum_{pqrs}h_{pqrs}a_{p}^{†}a_{q}^{†}a_{r}a_{s}$$

where we have partitioned the total Hamiltonian into its one-electron and two-electron terms, shown in eqs 4 and 5, respectively

4 $$h_{pq}=\int dx\phi_{p}^{*}\left(x\right)\left(-\frac{\nabla^{2}}{2}-\sum_{j}\frac{Z_{j}}{\left|r-R_{j}\right|}\right)\phi_{p}\left(x\right)$$

5 $$h_{pqrs}=\int \frac{dx_{i}\;dx_{j}\phi_{p}^{*}\left(x_{i}\right)\phi_{q}^{*}\left(x_{j}\right)\phi_{r}\left(x_{j}\right)\phi_{s}\left(x_{i}\right)}{\left|r_{i}-r_{j}\right|}$$

Solving the Schrödinger equation exactly for the second-quantized form on traditional computers is not feasible, however, due to the exponential number of states generated by the creation and annihilation operators.

With the Hamiltonian shown in eq 3, we can tackle the problem by employing a quantum computing algorithm. The current landscape for quantum computational chemistry can be roughly split into two algorithms and their variations, namely quantum phase estimation (QPE)^17^ and the variational quantum eigensolver (VQE).^14,30^ While QPE can be used directly to estimate the ground state energy from the phase, there have been additional works based on time series estimation.^18^

To date, there has been a plethora of works which have realized those algorithms on major quantum hardware architectures for a variety of molecular systems using both VQE^14–16,31,32^ and QPE.^32–36^ Recent work has presented an embedding scheme using QM/MM and bootstrap embedding,^37^ however, in place of a quantum computing algorithm, they used CCSD as a surrogate. Wave function in DFT projector-based embedding methods^38,39^ have also been proposed for use in the context of VQE^40^ and have recently been applied in the context of QM/MM.^41^ Another popular embedding methodology is density matrix embedding theory (DMET),^42,43^ which has been used alongside VQE^44,45^ and also used in a method for hybrid quantum-classical geometry optimization.^46^ Recently SqDRIFT has been developed^47^ to target quantum-chemical calculations. Combining existing work on qDRIFT^48,49^ and sample-based Krylov quantum diagonalization (SKQD),^50^ SqDRIFT extends the asymptotic scaling of qDRIFT with the convergence and efficiency of SKQD. As a result, this new method enables efficient sampling of wave functions. Additionally, the required circuits are generally shallower than those required for QPE making SqDRIFT especially suitable for early fault tolerant quantum computers.^47^

### Quantum Phase Estimation

Quantum phase estimation^17^ is used to find the phase or smallest eigenvalue for the system evolving under a targeted unitary operator. In the context of quantum chemistry, this unitary operator can be the electronic Hamiltonian, with the solution corresponding to the ground state energy of the system.

For the original (so-called textbook) version of QPE, to obtain the estimate of energy accurate up to *n* bits and with success probability *p*, Nielsen and Chuang^7^ have shown that one requires $n+\lceil \log_{2}\left(2+\frac{1}{2\left(1-p\right)}\right)\rceil$ ancilla qubits. As such, especially in the NISQ era, the qubit requirement is significant. Indeed, much work has been put into improving QPE, resulting in the development of iterative QPE, with only one ancilla qubit, where the phase is measured sequentially.^17,51^ For this work, we have benchmarked the iterative QPE variant described in detail by Somma.^18^

One caveat to utilizing QPE is that the initial state of the quantum register must have nonzero overlap with a desired ground-state.^10^ To fulfill this requirement, often a Hartree–Fock state is selected as an initial state of the quantum register.^10^ Moreover, the unitary operator must be an efficiently invertible function of the Hamiltonian. As such, the most common unitary operator is selected to be e^–i*Ht*^.^10^

However, to efficiently encode this operator in terms of single- and two-qubit quantum gates, one needs to decompose it via the Lie–Trotter–Suzuki approximation^52^ (in this work we only consider first-order approximation, although higher-order formulas have been studied^53–55^ ).

Unfortunately, this is another source of errors in the solution. In general, Reiher et al.^56^ have shown that the error in the obtained energy eigenvalue is upper bounded by errors stemming from the phase estimation algorithm, unitary decomposition and due to imperfect implementation of the gates during circuit synthesis.^10^ Further ways to optimize the resources and to minimize errors are an active area of research.^10,56,57^

### Fermion to Qubit Mapping

To solve the electronic structure on a quantum computer, one needs to encode the problem specified in second quantized form onto the device. Specifically, one needs to map from operators that act on indistinguishable electrons (Fermions) to operators that act on distinguishable qubits.^10^ In this work we consider Bravyi–Kitaev encoding,^58^ which has scaling advantages over the alternative encoding schemes.

Although a simpler encoding scheme, the Jordan–Wigner mapping, exists, it incurs the cost of *O*(*N*) Pauli operators acting on different qubits due to strings of *Z* operators. The Bravyi–Kitaev scheme^58^ scales as *O*(log_2_ *N*) in Pauli operators, instead. The idea behind this is that qubits store partial sums of occupation numbers, therefore storing both the partial information about the parity and occupation locally, given by

$$q_{p}=\left[\sum_{q=0}^{p}\beta_{pq}f_{q}\right]\;\mathrm{mod}2$$

where *f*_*q*_ is the occupation number stored in each qubit and scaled with Bravyi–Kitaev matrix which is defined recursively^58,59^

$$\beta_{1}=\left[1\right]$$

$$\beta_{2^{x+1}}=\left(\begin{array}{ll} \beta_{2^{x}} & 0\\ A & \beta_{2^{x}} \end{array}\right)$$

where *A* is (2^*x*^ × 2^*x*^) zero matrix with bottom row filled with 1’s and 0 is (2^*x*^ × 2^*x*^) zero matrix. The matrix is defined as a power of two in terms of qubits. If the number of qubits *N* is not an exact power of two, the next largest matrix is selected, and only the first *N* rows are used.^10^

Although the operators resulting from Bravyi–Kitaev encoding are more complex when compared to Jordan–Wigner mapping, the mapping yields better performance and reduces the required quantum resources.

### Workflow for Combined Quantum and Classical Computation

A workflow for combining conventional HPC calculations with quantum computing algorithms has been implemented in a development version of the Python-based QM/MM software package ChemShell.^26^ ChemShell acts as a generic driver that can seamlessly link multiple computational chemistry packages together to create complex workflows. In this paper, the workflow is demonstrated using a simulated quantum computer running on an HPC cluster, but the framework is intended to be generic so that either a simulator or quantum hardware may be used.

A simplified, diagrammatic representation of the workflow can be seen in Figure 1. The quantum computation is integrated into ChemShell by inheriting the base class common to all other (traditional) quantum chemistry packages (e.g., ORCA,^60,61^ TURBOMOLE,^62^ PySCF^63,64^ etc.). This modular approach ensures that the methodology can use features already present in ChemShell (i.e., QM/MM embedding, optimization methods, transition state determination etc.). In the first step of the workflow the user provides a definition of the molecule, defining properties such as geometry, charge, multiplicity and basis set. These parameters are read in by ChemShell, which then dispatches a call to a quantum chemistry package to determine the Hartree–Fock molecular orbitals; in this paper we use the PySCF package (however, other packages are usable, in principle). The quantum chemistry package then outputs a FCIDUMP file, which contains the terms of the electronic Hamiltonian (shown in eqs 3–5). In the next stage of the workflow, the quantum circuit is generated through a call to the external package Discover Lite, which uses the Hamiltonian terms from the previous stage to generate a circuit that implements the iterative QPE method.^18^ The circuit is generated in OpenQASM3.0 format.^65^ The ChemShell workflow then executes the circuit through a call to QASC, a component of the quantum integration software suite (QISS).^66^ QASC parses the circuit and interprets it by simulating the quantum program, thus, serving as a quantum backend. In the workflow every shot is parallelized using the message passing interface (MPI), allowing for the distribution of shots across an HPC cluster. The ChemShell workflow is agnostic as to whether the circuit is emulated or run on a quantum device, providing that the backend can accept an OpenQASM circuit as input and return a histogram of measurements.

**Figure 1:**
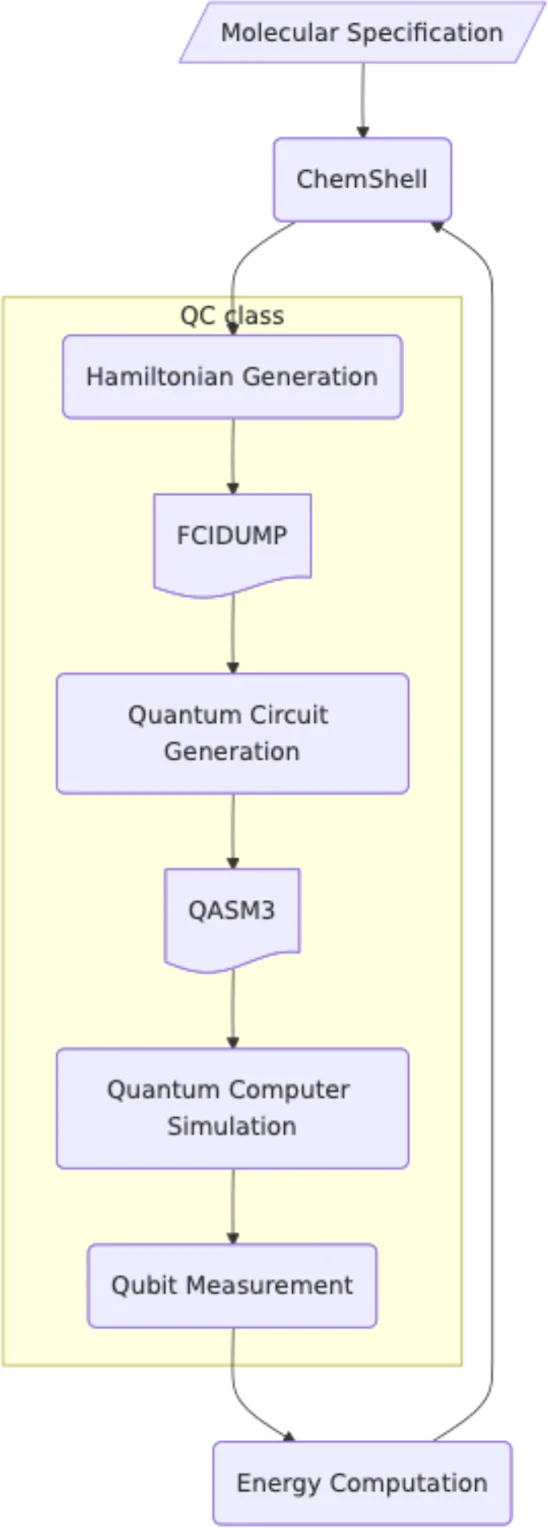
Outline of the workflow that integrates quantum computing algorithms with ChemShell. The steps inside the yellow box are all encapsulated within the same quantum computing class, which is derived from ChemShell’s quantum mechanics base class.

### Quantum Circuit Generation

The workflow generates a quantum circuit through a call to the external package Discover Lite, which takes Hamiltonian integrals in the FCIDUMP format generated in the first stage of the workflow and performs Fermion-to-Qubit mapping using Bravyi–Kitaev scheme. A perspective on approaches used in Discover Lite is available in ref 67. Given a desired accuracy, it computes the number and size of Trotter steps required for a single shot of QPE. It then computes the number of shots required to reach a desired probability of success. Finally, it generates the quantum circuit which implements iterative QPE and is written in OpenQASM3.0 ^65^ format. The rationale for using OpenQASM3.0 is that Iterative QPE requires midcircuit measurement with complex conditional logic for which OpenQASM2.0 ^68^ lacks capability.

### Quantum Circuit Simulation

The next stage of the workflow simulates the quantum circuit, involving a call to the external software QASC. The version presented in this work is an interpreter, combining functionality of a parser and quantum circuit simulation backend. The tool was developed in C++, ensuring performance and scalability.

QASC itself accepts an OpenQASM file (supporting both OpenQASM2.0 and OpenQASM3.0) representing quantum circuits as an input. Even though the parser component is generic, accepting a wide range of OpenQASM files, it is predominantly tailored toward circuits generated by Discover Lite from the previous stage of the workflow. The program parses the contents of the file, verifying validity of the circuit and generating data structures required for the simulation.

One issue arising from using older OpenQASM formats is the size of the quantum circuits. Given the problem domain and the current programming model for gate-based quantum computers, the quantum circuits grow to millions of gates. Because circuits written in OpenQASM2.0 are also valid OpenQASM3.0 circuits, support for large OpenQASM2.0 files is required. To enable this, files are parsed using a block buffer. The buffer would read-in and store chunks of the circuit, simulate it and then repeat the process until the whole circuit was executed. This provides the benefits of constant memory requirement, irrespective of the circuit size and the possible speedup in terms of I/O. Indeed, for meaningful problem sizes, the performance penalty of reading the file in this manner is negligible. However, in preliminary work we have identified that quantum circuits written in OpenQASM2.0 are inherently limiting as even for modest chemical systems the resulting temporary files occupy gigabytes of space. Yet, given the repetitive nature of the file and limited range of characters, such files could be readily compressible. This in turn might be of interest to the quantum compiler community. Due to this, in the ensuing work,^66^ we have focused on the OpenQASM3.0 format that introduced classical loop constructs to alleviate the problem, with resulting temporary files being in the range of tens of megabytes. The size is predominantly attributed to the approximations of the controlled unitary evolution required for QPE.

The quantum simulation component of QASC is built upon the QuEST library.^69^ This choice of software framework ensures the scalability and high-performance requirements for achieving our aim of simulating larger molecules. Additionally, QuEST allows for multithreading (with OpenMP), distributed computing (with MPI), and GPU accelerated computing (CUDA, cuQuantum, HIP, and Thrust), making it an ideal choice for simulated backends.

The quantum backend is responsible for the simulation of the exact (i.e., storing all intermediate parameters) statevector and density-matrix dynamics. This gives the user full control and complete information during the evolution of the state. However, due to the system’s quantum nature, the computational cost of simulation grows exponentially with system size in both memory usage and time to solution, limiting the size of molecule that can be studied. At the same time, the problem is embarrassingly parallel, and one can take advantage of available resources. Aside from the parallelization and distribution of the statevector simulation (for details about specific methods we refer to ref 69), an additional level of parallelism can be applied by running each shot in parallel during multishot execution. In this way, different simulations can be run concurrently as if there were *N* number of quantum devices.

In the shot-level parallelism, the identical quantum circuits are offloaded to different instances of workers and then sampled. Here we can achieve almost perfect linear scaling with respect to the number of shots per process, since the problem is embarrassingly parallel, and the only serial work is the creation and offloading of the circuits. Indeed, in the case of our workflow, the circuits generated by Discover Lite give clear requirements on the number of shots required to achieve the correct results with high probability, in this case, a 90% chance of correctness. In this situation we parallelize the shots by leveraging MPI processes which run concurrently. There is no communication bottleneck as the shots are independent. This strategy significantly speeds up the time-to-solution.

The output from this stage of the workflow is the collection of measurements resulting from the quantum circuit. These can be combined with follow-up functionality in Discover Lite to extract the ground state energy of the system based on the method presented in Somma.^18^ Once the energy is extracted in this way, it can be used for further chemical computations discussed in more detail in the next section.

### Multiscale Subtractive Embedding

Multiscale embedding schemes facilitate the use of multiple levels of theory within the same computation. One of the most well-known categories of multiscale embedding schemes is known as quantum mechanics/molecular mechanics (commonly abbreviated to QM/MM). In the QM/MM approach, a higher accuracy computation (QM) is embedded within a lower accuracy computation (MM), thereby using higher accuracy, but with much slower computation, only where required and lower accuracy, but with much faster computation, for the rest of the system. All embedding is performed atomically, meaning that the QM and MM regions could be described clearly as two sets of atoms. Broadly, QM/MM models fall into two categories: *additive embedding* and *subtractive embedding* schemes. In the additive embedding scheme, the intersection of the QM and MM atomic sets are mutually exclusive (disregarding link-atoms), whereas, in the subtractive scheme the QM atoms are a subset of the MM atoms (again, disregarding link-atoms). In this work, we chose to use the subtractive embedding scheme due to the relative simplicity of interactions between layers, compared to the additive scheme. The *subtractive embedding* scheme (alternatively known as the ONIOM scheme), was first introduced by Maseras and Morokuma.^70^ The scheme, in its most general form, allows for the recursive partitioning of molecular systems based on either orbitals or atoms. For clarity, we will first describe the subtractive embedding scheme for a two-layer partition and then generalize this onto an *N*-layer system, as implemented in ChemShell.

Consider an entire molecular system to be a set of atoms (*M*), which can be partitioned into two sets of atoms: an outer set (*O*) and an inner set (*I* = {*I*_c_, *I*_l_}), where *I*_c_ are the *core* atoms and *I*_l_ are the *link* atoms. The subtractive partitioning scheme requires: *I*_c_ ⊂ *O* and *I*_l_ ∩ *I*_c_ = ⌀. In the case where the outer region encompasses the entire molecular system (*O* = *M*), we can express the total energy as shown in eq 6

6 $$E\left(O\right)=E_{MM}\left(O\right)+E_{QM}\left(I\right)-E_{MM}\left(I\right)$$

The first two terms of eq 6 represent the total energy as the summation of the outer and inner regions and the final term corrects this summation, by removing the double-counted energies that result from (*I*_c_) being a subset of both *I* and *O*. In many cases, we are looking to also compute the forces associated with the subsets and the total system, care must be taken when there are link-atoms present (i.e., *I*_l_ ≠ ⌀), as these atoms are artifacts of the partitioning scheme and not in the actual molecular system (*O*). We will not discuss the link-atom corrections to the forces here as they have been discussed in detail elsewhere.^71^

To generalize the above two-layer case into the *N*-layer case, we can define a subset of the total system *L*_*i*_ and the methodology associated with the subset as *T*_*i*_. Given that *L*_*n*_ ⊂ *L*_(*n*+1)_ where 1 ≤ *n* < *N* and *L*_*N*_ = *M*; we can express the total energy in the *N*-layer case using the recursive relationship

7 $$E\left(L_{i+1}\right)=E_{T_{i+1}}\left(L_{i+1}\right)+E\left(L_{i}\right)-E_{T_{i+1}}\left(L_{i}\right)$$

Given an initial condition as the energy of the innermost layer

8 $$E\left(L_{1}\right)=E_{T_{1}}\left(L_{1}\right)$$

This recursive scheme indicates clearly that each layer has a dependence on the previous one. Though this recursion is conceptually useful, we can remove all recursion in the definition of the *N*-layer subtractive scheme energy and instead express it as the difference between two terms

9 $$E\left(L_{N}\right)=E_{addn}\left(L_{N}\right)-E_{subtr}\left(L_{N}\right)$$

where the additive term (*E*_addn_(*L*_*N*_)) and subtractive terms (*E*_subtr_(*L*_*N*_)) have the same interpretation as eq 6, where the additive terms sum the energy of the two regions and the subtractive term corrects for the double counting of the layer in the next summation term. The additive and subtractive terms can therefore be expressed simply as two summations and are shown in eqs 10 and 11, respectively.

10 $$E_{addn}\left(L_{N}\right)=\sum_{i=1}^{N}E_{T_{i}\left(L_{i}\right)}$$

11 $$E_{subtr}\left(L_{N}\right)=\sum_{i=1}^{N-1}E_{T_{i+1}\left(L_{i}\right)}$$

To simplify the approach in ChemShell, we introduced a “Layer” object, which couples and manages *T*_*i*_ and *L*_*i*_, for each *i* (including all link-atoms and their parameters). This Layer object is then used to apply the energies, shown in eqs 10 and 11, and the corresponding forces.

### Active Space Selection in Embedded QPE

Due to the computational cost of simulating quantum computations on HPC hardware and the limitations on the number of viable qubits on a quantum computer, it is necessary to reduce the number of electrons and orbitals required in the quantum computation. One method by which we can do this is by choosing an active space over which to perform the quantum computation. An active space set of orbitals is a subset of the total orbital space in a molecular system, the purpose behind choosing an active space is to reduce the computational cost by reducing the problem size. The chosen active space should target orbitals that would benefit the calculation most if treated beyond the mean-field approach (in this case Hartree–Fock), or select the orbitals most applicable to the chemistry one wishes to study. In this paper, we use the term active space to denote the set of orbitals that the quantum computing algorithm (QPE) is applied to.

In this section, therefore we employ an automated method for computing the active space of a molecule, based on previous work;^72,73^ formally, it is most similar to Izsák et al.^73^ Other methods, such as atomic valence active space (AVAS)^74^ and unrestricted natural orbital (UNO)^75^ also exist. With respect to AVAS, the approach here shares similarities in that occupied orbitals are selected by maximizing the overlap with a minimal basis, used to represent the region of interest. The region of interest in our case is a set of atoms; in the case of AVAS it is a choice of valence orbitals. The method here shares similarities with UNO in the use of MP2 orbital populations to determine which orbitals to include in the active space. Given that we use a numerical cutoff to determine the orbitals for the active space calculation, the method described here could suffer from discontinuities in the potential energy surface, if new orbitals are chosen at each time step; this could be mitigated by only considering the same set of orbitals over the geometry change.^76,77,78^ To build an active space that represents the local region of interest in the molecule, we first localize the occupied orbitals and then project the localized orbitals onto a minimal basis representation of the region of interest. This projection allows for the quantification of the overlap between the localized-occupied orbitals and the region of interest. A cutoff can be applied to the overlap values such that localized-occupied orbitals which do not strongly overlap with the fragment of interest are frozen (and therefore will not be part of the resultant active space).

We then run an MP2 calculation, on the remaining subspace of nonfrozen occupied-localized orbitals and all virtual orbitals. At no stage are the virtual orbitals localized. The diagonal elements of the MP2 first-order-reduced density matrix are the natural orbital (NO) occupancies. We then select the occupied NOs with the lowest occupancies and the virtual NOs with the highest occupancies. By computing the occupancies of the natural orbitals, it is possible to separate those which are most likely to be part of the *core* set of orbitals (i.e., well represented by the Hartree–Fock ground state) and those that are not well represented by the Hartree–Fock state. This is the automatically determined active space. The steps for determining the active space are listed in Scheme 1.

**Scheme 1:**
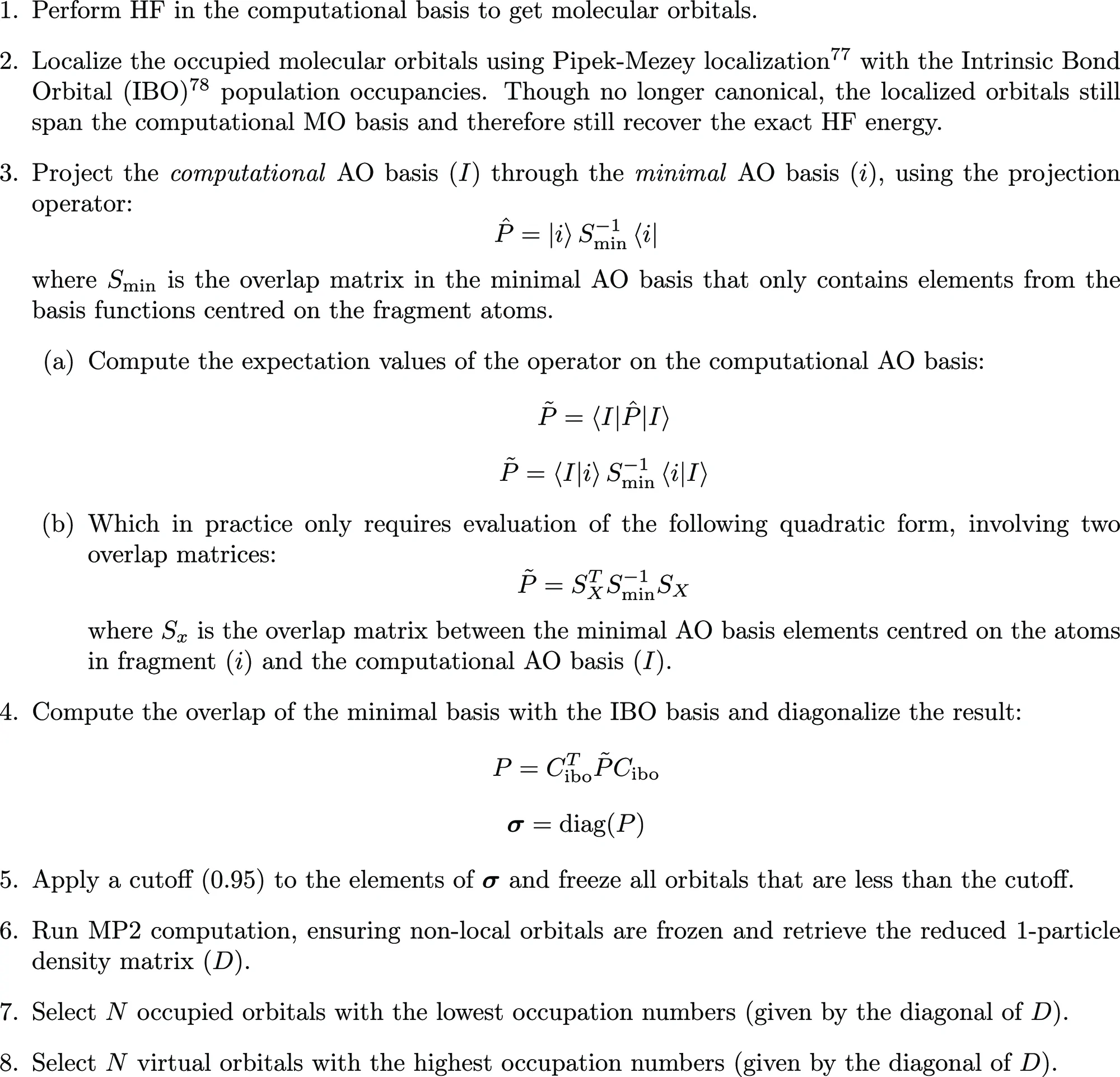
Steps Required to Automatically Determine the Active Space Orbitals over a Molecular Fragment

Steps 7 and 8 in Scheme 1 provide the set of active space orbitals that will be embedded into the system using the following subtractive embedding

12 $$E_{total}=E_{total,CCSD}-E_{active‐space,CCSD}+E_{active‐space,QPE}$$

This embedding can be used along with the previously described atomistic multiscale subtractive embedding, allowing for large scale computations to be performed including a tractable quantum computing region. This results in a 4-layer embedding as shown in Figure 2.

**Figure 2:**
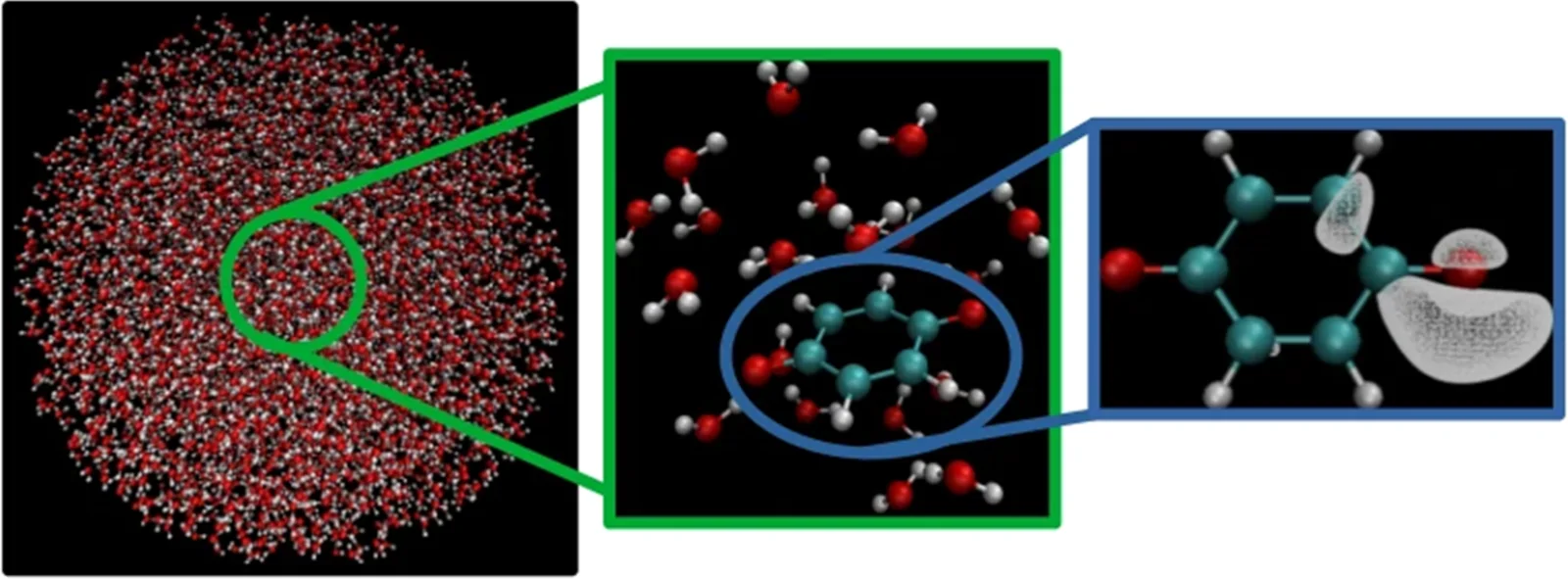
Illustration of the 4-layer embedding scheme. The first panel shows the entire system, computed using MM; the second panel shows the region computed using DFT, which contains paraquinone and 20 water molecules; and the third panel shows paraquinone molecule, computed at CCSD/def2-SVP and one of the active space orbitals (QPE/def2-SVP). All molecules and orbitals were visualized with VMD.^79^

### Computational Details

Benchmarks were run for three sets of systems of increasing complexity: first, a series of small molecules were run using QPE without embedding; second, a water droplet, with QPE embedded in a QM/MM environment; and third, a two-layer QPE/CCSD embedding calculation, again embedded in two further QM/MM layers. All QPE computations were run to an accuracy of 0.01 Ha.

### Small Molecule Series

Initially, we studied a selection of small molecular systems to benchmark the quantum simulation running on CPU and GPU HPC systems. Unless otherwise stated, the CPU and GPU hardware used in this work were 32 Intel­(R) Xeon­(R) Gold 6142 CPU@2.60 GHz and NVidia V100s, respectively. The molecules chosen are closed shell systems with no overall charge, with elements drawn from the first and second periods of the periodic table. These molecules were chosen to understand the trend in performance when the size of the molecules (and therefore orbital space) increases. Table 1 gives information regarding each molecular system. PySCF generates an FCIDUMP file to be used in building the quantum circuits; Hamiltonian terms with absolute magnitudes less than 1 × 10^–15^ were excluded from the FCIDUMP file. Each of the quantum circuits were generated using Discover Lite, from which the logical circuit size was measured in terms of depth (with respect to both single qubit and 2 qubit gates). Given all the molecular systems are closed shell, the number of orbitals has been expressed in terms of spatial orbitals, not spin orbitals, where the latter is formed as the product of the former with a either an α or β spin function. There are therefore twice as many spin–orbitals as spatial ones.

**Table 1:** Computational Details of the Single Molecule Benchmarks^a^

	electrons (*N*)	spatial orbitals (K)	Hamiltonian terms (after filtering)	circuit depth	circuit depth (2 qubit gates)	number of qubits
H_2_	2	2	8	9180	6120	3
LiH	4	6	189	47,878,740	34,398,000	11
BeH_2_	6	7	363	175,139,962	127,353,668	13
BH_3_	8	8	996	1,245,697,788	907,093,944	15
NH_3_	10	8	924	1,244,321,616	906,438,624	15
H_2_O	10	7	347	350,301,306	254,722,884	13
CH_4_	10	9	2070	2,894,941,632	2,122,843,608	17

a All information was generated using HF/STO-3G. Presented depths refer to the logical quantum circuits, not taking into account quantum error-correction overheads.

### Three-Layer QPE/DFT/MM

A water droplet model was used to benchmark QPE calculations embedded into an HPC QM/MM environment using the subtractive embedding scheme. This system consists of 5615 water molecules in total. The system was a pre-equilibrated box of water using the TIP3P force-field, this box was then truncated to create a roughly spherical “droplet”. The layers were partitioned by atomic indices: the innermost layer contains only a single water molecule; the middle layer contains 20 water molecules; and the outer layer contains all 5615 water molecules. The inner layer computation was performed at QPE/STO-3G level of theory; the middle DFT layer at the B3LYP/6-31+G­(d,p) level of theory and the outer layer using the classical force-field TIP3P.^80^

### Four-Layer QPE/CCSD/DFT/MM

The full embedding scheme including active orbital space embedding has been benchmarked using a paraquinone molecule solvated in a water droplet. We have used an automated orbital selection method for paraquinone as outlined in the methods section. The selected molecular fragment over which to build the active space was a single C=O moiety. This results in a 4-layer embedding, where the innermost layer is a selection of active-space orbitals, the smallest being (4, 3) and the largest (10, 9), where the first number is the number of electrons and the second is the number of *spatial orbitals*. This layer is computed at the iterative QPE level of theory and shares the same basis set as the second innermost layer. The second innermost layer calculates the full paraquinone molecule, computed at the CCSD/def2-SVP level of theory. The third layer adds a further 20 water molecules around paraquinone and is computed at the B3LYP/6-31+G­(d,p) level of theory. The outermost layer is described using classical molecular mechanics, adding the remaining 5587 water molecules of the droplet model. The MM calculation on the full system uses the TIP3P force-field for the water molecules and the GAFF2 force-field for the paraquinone molecule.

## Results

### Small Molecule Series

Table 1 shows the details of the small molecule series computations. We have captured a range of system sizes, from 3 to 17 qubits, 8–2070 Hamiltonian terms and a range of OpenQASM file sizes from 77 to 385,853 lines, in line with what can be feasibly simulated on HPC systems.

Table 2 shows the (per shot) computational timings of the small molecules for both CPU and GPU. Graphical representations of the CPU and GPU benchmarks are presented in Figures S1 and S2, respectively. The classical preprocessing time includes the generation of the HF Hamiltonian and all associated I/O (i.e., writing the relevant Hamiltonian terms to the FCIDUMP file). The circuit generation column includes the time required to compute and write the circuit file to disk. The circuit simulation column was the time taken to simulate the quantum circuit on either the CPUs or GPU. It is important to note that this step has been parallelized across multiple MPI ranks and the time reported is the time required by a single shot on a single rank. In other words, if all 6 shots (as were run in this case) were serialized, the total runtime for this column would be approximately 6 times the value reported.

**Table 2:** Computational Timings of Each of the Small Molecule Computations Described in Table 1 ^a^

	CPU				GPU				
	classical preprocessing time/s	circuit generation (per shot)/s	circuit simulation (per shot)/s	total time/s	classical preprocessing time/s	circuit generation (per shot)/s	circuit simulation (per shot)/s	total time/s	computational cost (of using GPU over CPU) (%)
H_2_	0.2	6	13	19	5	7	3	15	76
LiH	0.2	13	605	618	7	12	184	202	33
BeH_2_	0.2	27	2761	2789	1	22	724	747	27
BH_3_	7.9	433	40,271	40,712	4	205	6399	6608	16
NH_3_	0.2	263	40,213	40,476	2	199	6252	6454	16
H_2_O	0.2	27	5586	5614	5	23	1424	1452	26
CH_4_	#N/A	#N/A	#N/A	#N/A	6	1133	23,474	24,614	#N/A

a The total time is broken down into the classical preprocessing time, the circuit generation time and the quantum circuit simulation time. The computational cost for the classical computation on CPU for the CH_4_ molecule were too computationally expensive to run within scheduling limits, and have been marked #N/A.

### Three-Layer QPE/DFT/MM

Benchmarks for the three layer system are presented in Table 3. The classical molecular mechanics layer takes the least amount of time to calculate, with the additive component taking *32 s* and the subtractive component taking *0.04 s*. The DFT layer took the second least amount of time, with the additive component taking *297 s* and the subtractive component taking approximately *48 s*. The innermost layer took the most amount of time, with the quantum computation taking *8350 s* on GPU and *20,092 s* on CPU; it should be noted that this is much more computationally expensive than that reported in Table 2, as we discuss below.

**Table 3:** Details of the 3-Layer Water-Droplet System, Including the Level of Theory Used in Each of the Layers, the Resultant Energies and the Timings of Each Layer^a^

layer	theory	size (# of water molecules)	addition energy/Ha	subtraction energy/Ha	addition time/s	subtraction time/s
inner	QPE/STO-3G	1	–75.015	–76.395	8350 (GPU)|20,092 (CPU)	48
middle	B3LYP/6-31+G(d,p)	20	–1528.035	–0.148	297	0.04
outer	TIP3P	5615	–74.701	#N/A	32	#N/A

a The inner layer is embedded in the middle layer, which is embedded in the outer layer.

The total energy of the system is shown below, where it has been decomposed into the relevant additive and subtractive components, all values are in hartrees. The QPE energy correction to the DFT energy is *+1.3803 hartrees*.

$$\left(\overset{QPE\left(0\right)}{\overset{︷}{-75.0151}}-\overset{DFT\left(0\right)}{\overset{︷}{-76.3954}}\right)+\left(\overset{DFT\left(1\right)}{\overset{︷}{-1528.0351}}-\overset{FF\left(1\right)}{\overset{︷}{-0.1482}}\right)+\overset{FF\left(2\right)}{\overset{︷}{-74.7014}}=\overset{total\;energy}{\overset{︷}{-1601.2078}}$$

### Four-Layer QPE/CCSD/DFT/MM

In the four-layer embedding system, two inner layers are computed with active-space embedding and the surrounding layers are described using subtractive QM/MM.

Table 4 presents timings for the layers described using conventional QM and MM calculations. As in the 3 layer model, the outer layer described using classical molecular mechanics is the fastest layer to compute and the outer–middle layer containing the 20 water molecules and paraquinone at DFT/6-31+G­(d,p) is the most expensive to compute and the inner–middle layer, that contains the paraquinone molecule at CCSD/def2-SVP, is the second-most expensive, taking *51 s*.

**Table 4:** Computational Details of the 3 Outermost Layers of the 4-Layer Embedding^a^

layer	theory	size (# water molecules)	addition energy/Ha	subtraction energy/Ha	addition time/s	subtraction time/s
inner–middle	CCSD/def2-SVP	0	–379.985	–381.228	51	13
outer–middle	DFT/B3LYP/6-31+G(d,p)	5	–1909.178	–0.450	854	0.03
outer	GAFF2/TIP3P	5607	295.238	#N/A	24	#N/A

a These benchmarks were run on an intel i7-12700H CPU. The information regarding the innermost layers (QPE) with different active spaces is shown in Table 5.

We have compared several active space sizes runs using simulated QPE as shown in Table 5. The (4, 4) active space example is shown in Figure 3. The computational cost of simulating QPE generally increases as the active space increases, with a roughly exponential trend with respect to the number of orbitals in the active space, as shown in Figure 4.

**Table 5:** Timings and Energies for Each of the Paraquinone Active-Space Sizes Studied^a^

number of electrons	number of (spatial) orbitals	circuit simulation (per shot)/s	QPE active space energy/Ha	CCSD active space energy/Ha	inner subtractive correction/Ha	total (4-layer) energy/Ha
4	3	198	–378.7978	–378.7977	0.0001	–1612.2473
4	4	305	–378.8309	–378.8273	0.0036	–1612.2438
6	5	507	–378.8332	–378.8275	0.0057	–1612.2417
6	6	1752	–378.8641	–378.8703	–0.0062	–1612.2536
8	7	1317	–378.8683	–378.8734	–0.0051	–1612.2525
8	8	4851	–378.9026	–378.9032	–0.0007	–1612.2481
10	9	11,196	–378.9059	–378.9039	0.0020	–1612.2454

a The values in this table are shown graphically in Figures 4 and 5.

**Figure 3:**
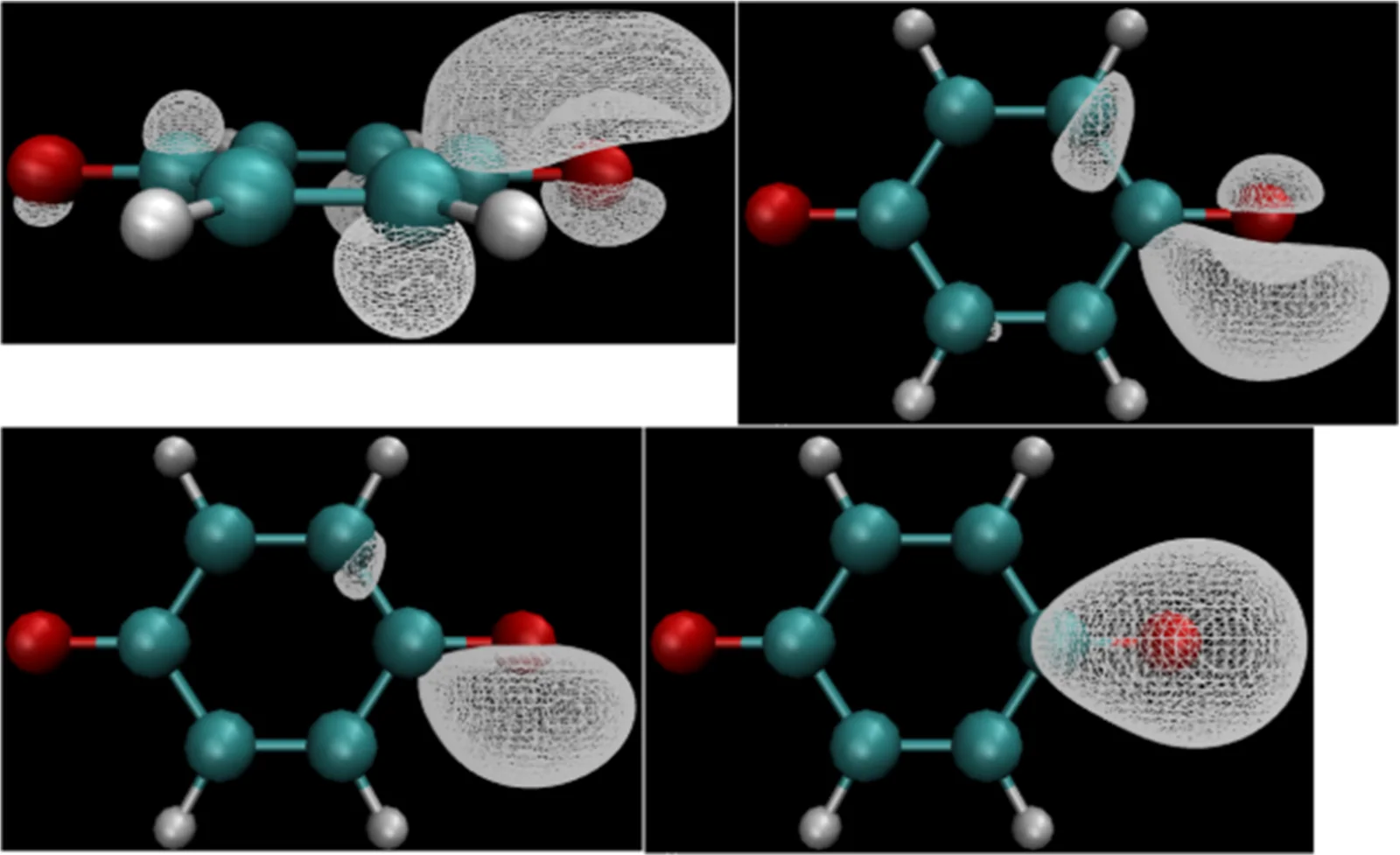
Active space orbitals of the (4, 4) paraquinone active space computed using the def2-SVP basis. These orbitals were selected using the active-space embedding selection scheme described in the methods. The top are the 2 virtual orbitals and the bottom row are the occupied orbitals. The orbitals were visualized using VMD.^79^

**Figure 4:**
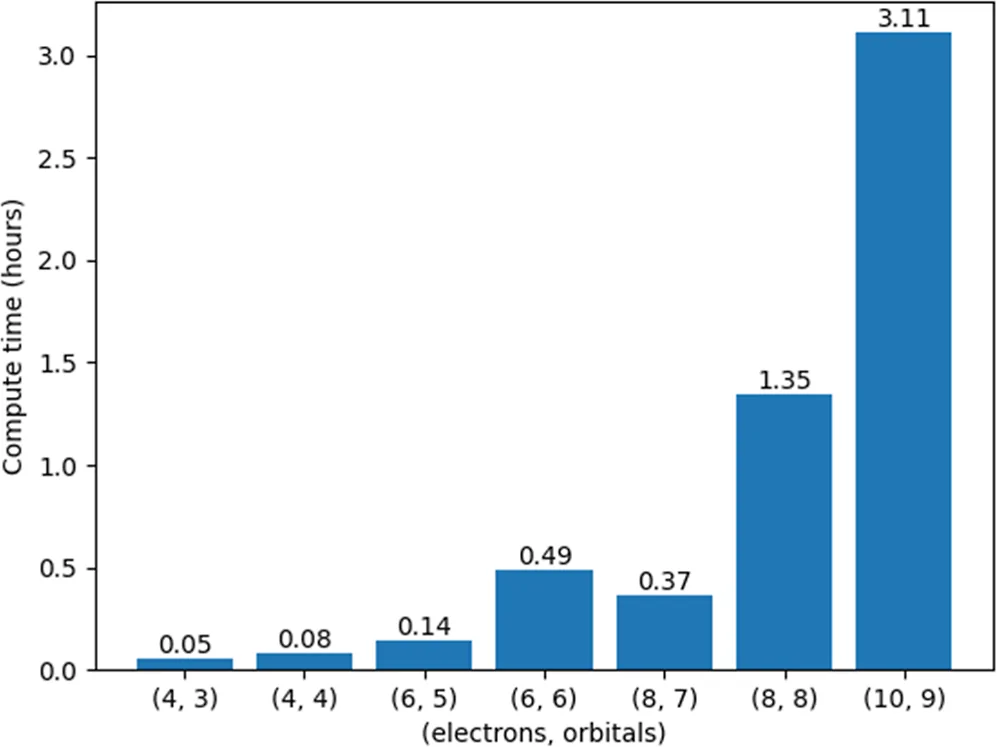
Shows the timings for the paraquinone active spaces. On the *x*-axis, the first number is the number of electrons in the active space and the second is the number of spatial-orbitals. All tests were run on Nvidia V100s. This graph is tabulated in Table 5.

Calculated QPE energies are plotted in Figure 5, where it is evident that the computed energy of the system decreases as the active space increases in size. This decrease in energy appears to be in a stepwise manner, where the biggest decreases in energy are observed when keeping the number of electrons the same but increasing the number of spatial orbitals by 1.

**Figure 5:**
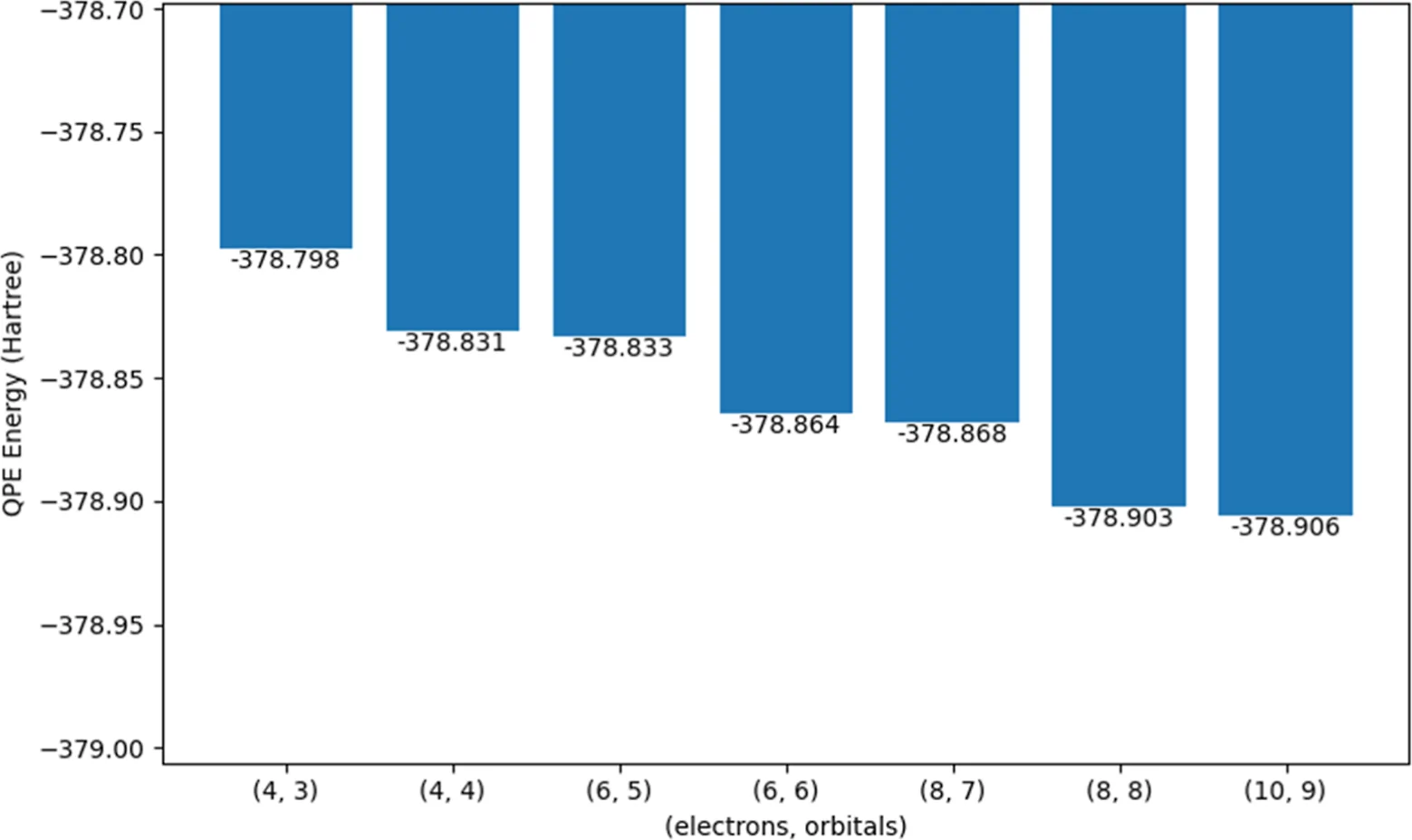
Shows the energies of the paraquinone active spaces. On the *x*-axis, the first number is the number of electrons in the active space and the second is the number of spatial-orbitals. All tests were run on NVidia V100s. This graph is tabulated in Table 5.

Table 5 gives details of the QPE/CCSD embedding calculations, including the subtractive scheme correction of the CCSD energy by the QPE active space. For this system the correction is usually around a *milli-Ha* order of magnitude, or less.

## Discussion

### Small Molecule Series

The timings given in Table 2 show that quantum circuit simulation is the most computationally expensive part of the calculation and grows exponentially as the system size increases. This exponential increase in computational cost is expected as the simulation of quantum computation, in general, increases exponentially as a function of the number of qubits. This exponential growth in computational cost is partially explained by the required size of the state-vector: which is of size 2^*N*^, where *N* is the number of qubits. The relationships of both the number of qubits and the size of the state vector with the computational cost are shown in Figure S3, where the exponential cost can clearly be seen. The second observation is that the circuit generation takes the second longest amount of computational time. Finally, the classical preprocessing time takes the shortest amount of computational time, the scaling of classical computation of Hartree–Fock is well-known to be polynomial and discussed elsewhere.

From Table 2, we can compare the cost of circuit simulation on CPU vs GPU. It is evident that circuit-simulation on GPU is much faster than on CPU. A graphical representation of the performance improvement is given in Figure S4. The largest performance increase seen on GPU is for the systems NH_3_ and BH_3_, where the GPU only takes about 16% of the total runtime when compared to CPU. From the perspective of memory requirements, it is expected to see significant benefit of offloading to GPU until a system saturates the memory of a single unit. Given the characteristics of the statevector simulation, simulating larger systems would require intense interdevice communication (multi-GPU statevector simulation), limiting scaling. However, based on the presented results and corresponding time-scaling of simulated QPE (including time-evolution operators), frequent simulation of such systems is unlikely and as such we highly recommend offloading to GPUs whenever possible.

### Three-Layer QPE/DFT/MM

When considering Table 3, it is evident that the layers with the greatest number of atoms took less time than the layers with the least number of atoms. The computational cost of each of the layers can therefore be attributed entirely to the scaling of each of the methods. The outermost layer uses molecular mechanics, which is linear with respect to the number of atoms (assuming a constant cutoff radius), DFT scales cubically with respect to the number of atoms, and on classical computers QPE simulation scales exponentially with the number of atoms, due to the exponential growth of the state vector.

When comparing Tables 2 and 3, we can see that the computational cost of circuit simulation for the water molecules is very different for each, even though both were computed at the same level of theory and with the same basis set (HF/STO-3G). The reason behind this difference in computational cost is due to the difference in the number of surviving Hamiltonian terms in the FCIDUMP file after applying the energy cutoff (*1 × 10* ^*–15*^). In the first case, the single water molecule in the gas phase, we find that ∼300 Hamiltonian terms are within the energy cutoff, compared to the ∼500 Hamiltonian terms that are retained within the same cutoff for the water module in the 3-layer system. The remaining terms in the Hamiltonian require an increase in the number of Trotterisation steps by a factor of 2, to stay within error bounds. This in turn increases the size of the circuit from 40,473 gates to 79,449 gates. The reason behind this increase in Hamiltonian terms with values *>1 × 10* ^*–15*^ appears to be due to the lack of geometric symmetry of the water molecule in the case where the water molecule is in a spherical droplet. Symmetry breaking is unavoidable in any complex chemical system and the results show that care must be taken when extrapolating performance insights from highly symmetric small molecule cases.

### Four-Layer QPE/CCSD/DFT/MM

The use of an active space allows larger molecules to be treated with a QPE calculation on a subset of orbitals, while introducing an additional complication of choice of active space. The observations made with respect to Figure 5 can be explained by understanding the active-space approach as a correction to the Hartree–Fock energy; the reason the energy decreases as the active-space decreases is then easily explained as the Hartree–Fock energy is always an upper bound on the *true* energy of the system. The active-space subsystem therefore captures more of the electron-correlation energy as the size increases; therefore the energy decreases. This decrease of energy with the increasing active space is an expected observation; this observation provides some evidence to support the fact that the automated active space algorithm is stable.

In this study, we used the Hartree–Fock methodology to generate the initial state from which the active space orbitals were determined. This is the approach most commonly used but note that the generation of initial states for QPE is a current area of active research.^81^

### General Considerations

In the current workflow, we are unable to compute the analytic gradients from the quantum computation. To approximate gradients using finite difference in the context of simulated quantum algorithms was computationally prohibitive, due to the exponential cost of increasing the accuracy of the QPE and due to the additional energy computations involved. In this work, we therefore focused on single point energies.

The benchmarks illustrate the limitations of using conventional HPC technology to simulate quantum circuits, as these timings unavoidably scale exponentially with system size. Nevertheless, they show that the framework can successfully be used to embed QPE calculations in a larger environment to describe chemical systems of much greater complexity than can be tackled using (simulated) QPE alone. Having established the overall framework, QPE calculations using quantum hardware can be incorporated as the hardware develops, and the size of the “quantum computing region” enlarged as the number of qubits available permits. Our use of emulated QPE to demonstrate the workflow is a proof of concept, which does not offer advantages for production calculations, but illustrates the potential for coupling HPC and quantum devices in a multiscale framework.

Analyzing the results of this work, it is clear that to run proposed workflows it is crucial to have access to advanced fault tolerant quantum computers capable of running millions of gates. Based on the recent analysis,^82^ devices with required capabilities could be expected by mid 2030s. Nevertheless, predicting state of the technology far to the future is notoriously challenging, and indeed, we should expect shifts in the rate of progress and roadmaps of different research groups.

At the same time, work such as presented in ref 47 highlights that we can expect further algorithmic developments that can reduce the requirements on the quantum resources and possibly deliver quantum utility sooner.

The workflow described in this publication will be made available as part of the Python-based version of ChemShell, which is available for download under the open source GNU LGPL v3 license from www.chemshell.org.

## Conclusion

In this work, we have implemented and benchmarked a multiscale workflow in ChemShell that can be used to embed high accuracy electronic structure calculations on a quantum computer within a lower accuracy quantum/classical environment calculated on traditional HPC systems. The work done here was performed by simulating the quantum computations, where we found that GPU simulation outperformed CPU simulation with respect to runtime of the QPE algorithm. We were also able to increase parallelism by distributing the shots across GPUs using MPI.

When considering a series of small molecules, we found that the computational cost of QPE increases with the size of the system and exponentially with the number of qubits required. We were also able to show that performing QPE with a GPU could cut the runtime down to 16% of the original CPU runtime.

In the 3-layer embedding system, we demonstrated the ability for the ChemShell framework to embed a QPE layer within two outer layers (DFT and MM, respectively). Despite the small size of the QPE layer (a single water molecule); we found that it was the most computationally expensive to run on both CPU and GPU, albeit with a large performance increase on the GPU. We found large performance differences between the water molecule at the energetically minimum geometry (in the small molecule series) compared to a geometry out of equilibrium (in the 3-layer water box). This performance difference was attributed to the number of elements in the Hamiltonian that were larger than the cutoff tolerance and not removed for the higher energy geometry in the nonequilibrium structure compared to the equilibrium structure. It is therefore important to choose the largest cutoff sensible for the Hamiltonian terms to obtain better performance for quantum computation.

In the final, 4-layer computation, we presented an embedding model that incorporated both atomistic and active-space embedding. The choice of active space was automated to focus on a pair of atoms. Using this approach, we were able to further restrict the QPE size, allowing for much faster computation. We also studied a range of active space sizes, showing exponential computation requirements as the active space size increased.

In the future, we would look to build on the work presented here in several ways. The first is to benchmark the quantum algorithms on a quantum computer, instead of a simulator, and so overcome the exponential scaling of the QPE region observed with simulated calculation. The embedding framework has been designed to be entirely generic, in line with ChemShell’s overall design, so that either simulation or quantum hardware can be used depending on what platforms are available. The quantum computing workflow within the embedding framework is also intended to be generic with respect to the quantum algorithm used. In future work, we will enable support of other quantum algorithms in addition to QPE, including other standard methods such as VQE and recent advances. We aim to extend the capabilities of our solution to accommodate new algorithmic developments, and enable energy estimation with alternative methods, targeting (early) fault-tolerant quantum computers.

The second is to address the challenges associated with improving the embedding schemes, for example, with the addition of electrostatic embedding. The third would be to compute analytic forces from the quantum computation, thereby allowing for more complex tasks, such as geometry optimization and molecular dynamics, it should be noted both these tasks are trivial to run in the ChemShell framework but would be challenging at present due to the necessity of calculating gradients using numerical finite-differences.

## Supplementary Material





## Notes

An archive containing ChemShell input files and molecular geometries for the small molecule, 3-layer and 4-layer benchmark systems (ZIP)
A PDF with additional figures illustrating the relative performance for each stage of the framework using CPU and GPU execution (PDF)
