# Supplementary material for: Hybrid HPC and Quantum Computing Framework for Multiscale Modeling of Chemical Systems
Source: J Chem Theory Comput. 2026 Aug 5;22(16):8201–14. doi: 10.1021/acs.jctc.6c00542 (PMC13523702; doi:10.1021/acs.jctc.6c00542)
Supplement: Supplementary file 1 [file ct6c00542_si_002.pdf]

Supporting Information

Hybrid HPC and quantum computing framework for multiscale modelling of chemical systems

Joseph C.R. Thacker,[†] Mateusz Meller,[‡] Alberto Popescu,[‡] Andrew Patterson,[§] Róbert Izsák,[§] Vendel Szeremi,[‡] and Thomas W. Keal[*,†]

† STFC Scientific Computing, Daresbury Laboratory, Keckwick Lane, Daresbury, Warrington, WA4 4AD, UK.

‡ STFC Hartree Centre, Daresbury Laboratory, Keckwick Lane, Daresbury, Warrington WA4 4AD, UK.

§ Riverlane Ltd., Cambridge CB2 3BZ, UK.

** Corresponding Author: thomas.keal@stfc.ac.uk*

Comparison of CPU and GPU Performance

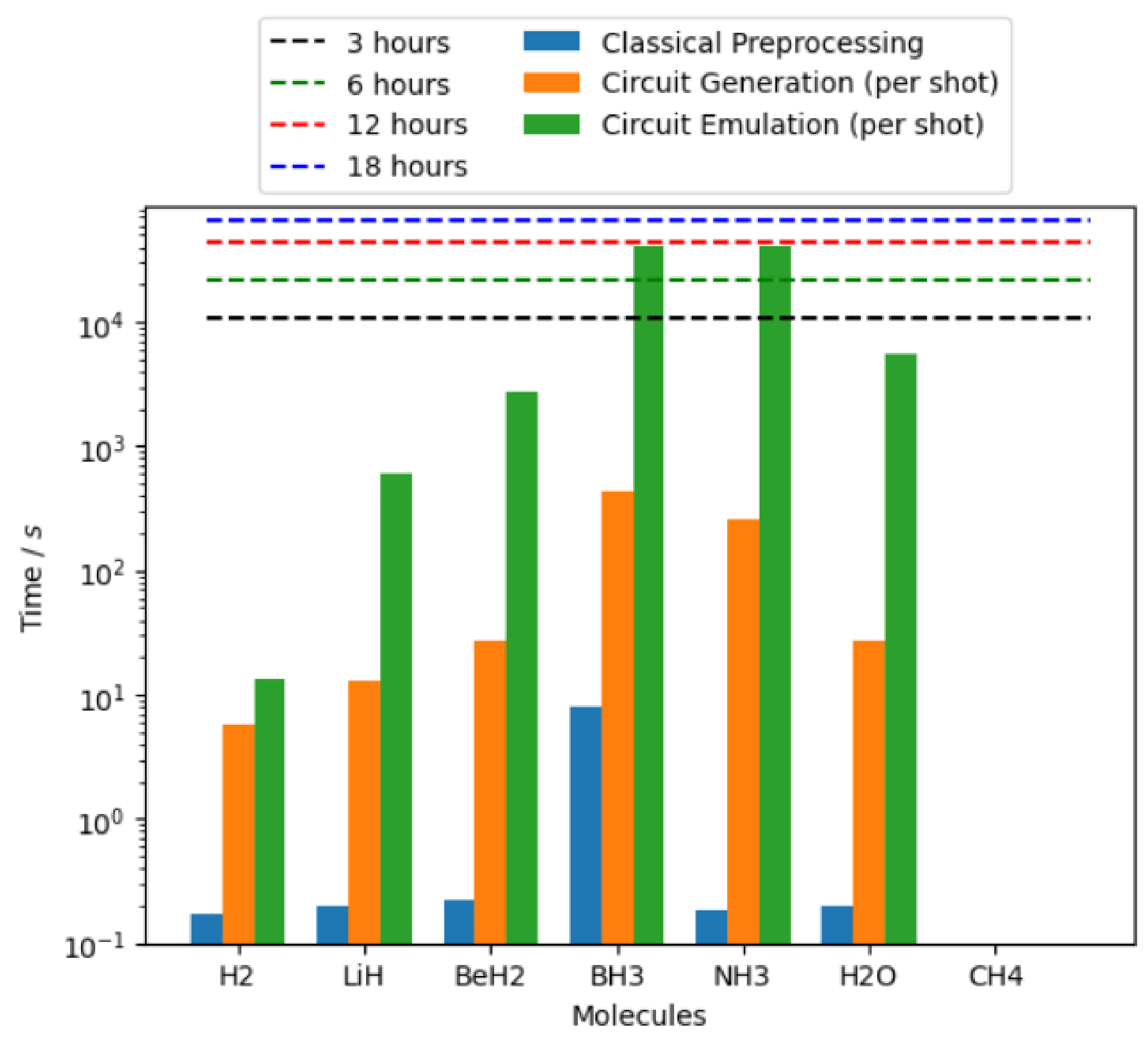

Figure S1. Computation time for each stage of a single-point energy QPE calculation for each molecule in the small molecule series. The circuit emulation time is only given for a single shot, as each shot can be run independently (i.e. embarrassingly parallel) using MPI. Circuit emulation was run using 32 CPUs per shot: Intel(R) Xeon(R) Gold 6142 CPU @ 2.60GHz. CH_4 data is not available due to inhibitive computational cost.

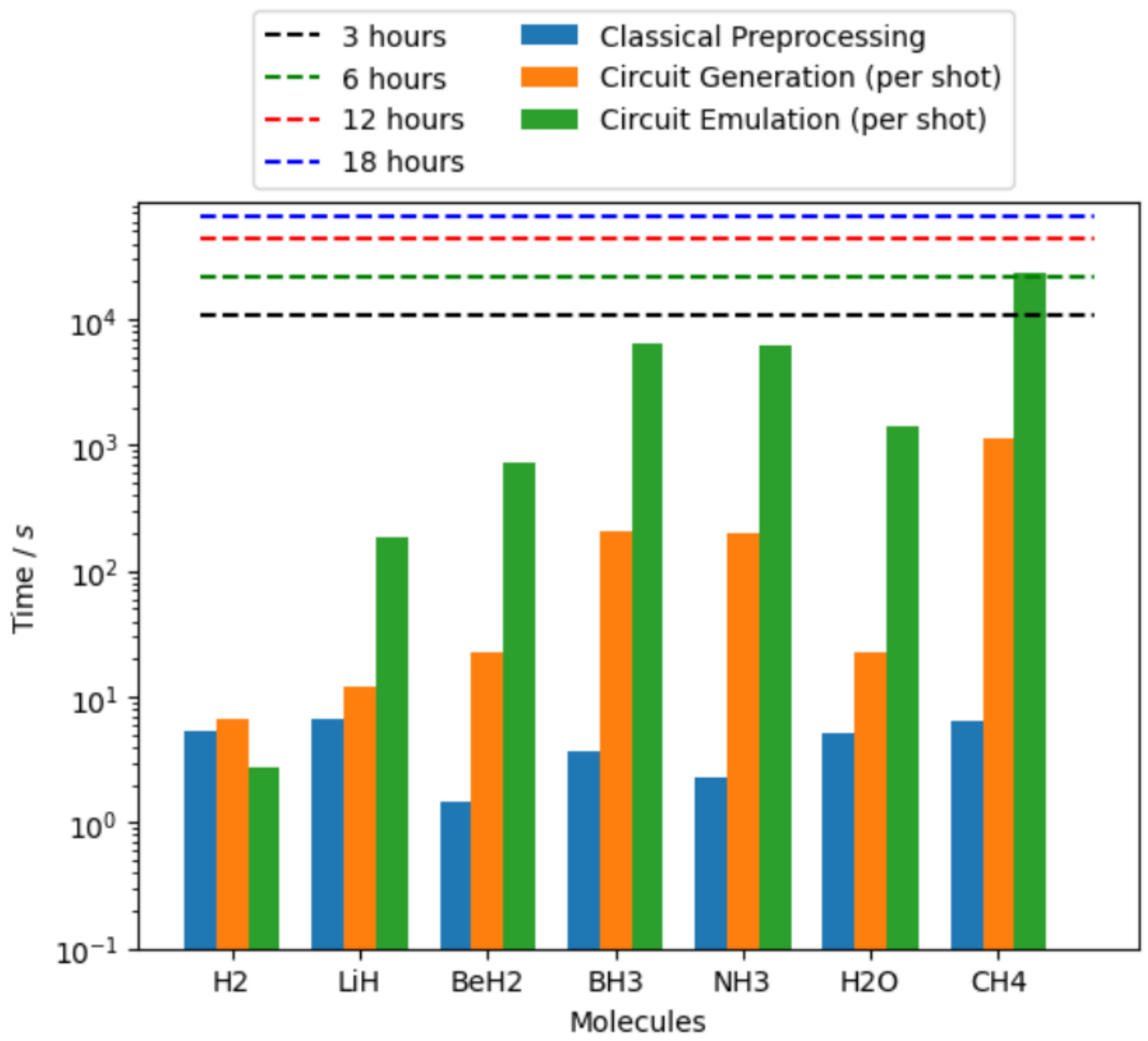

Figure S2. Computation time for each stage of a single-point energy QPE calculation for each molecule in the small molecule series. The circuit emulation time is only given for a single shot, as each shot can be run independently (i.e. embarrassingly parallel) using MPI. Circuit emulation was run using a single NVIDIA V100 per shot. All other steps were run on CPU.

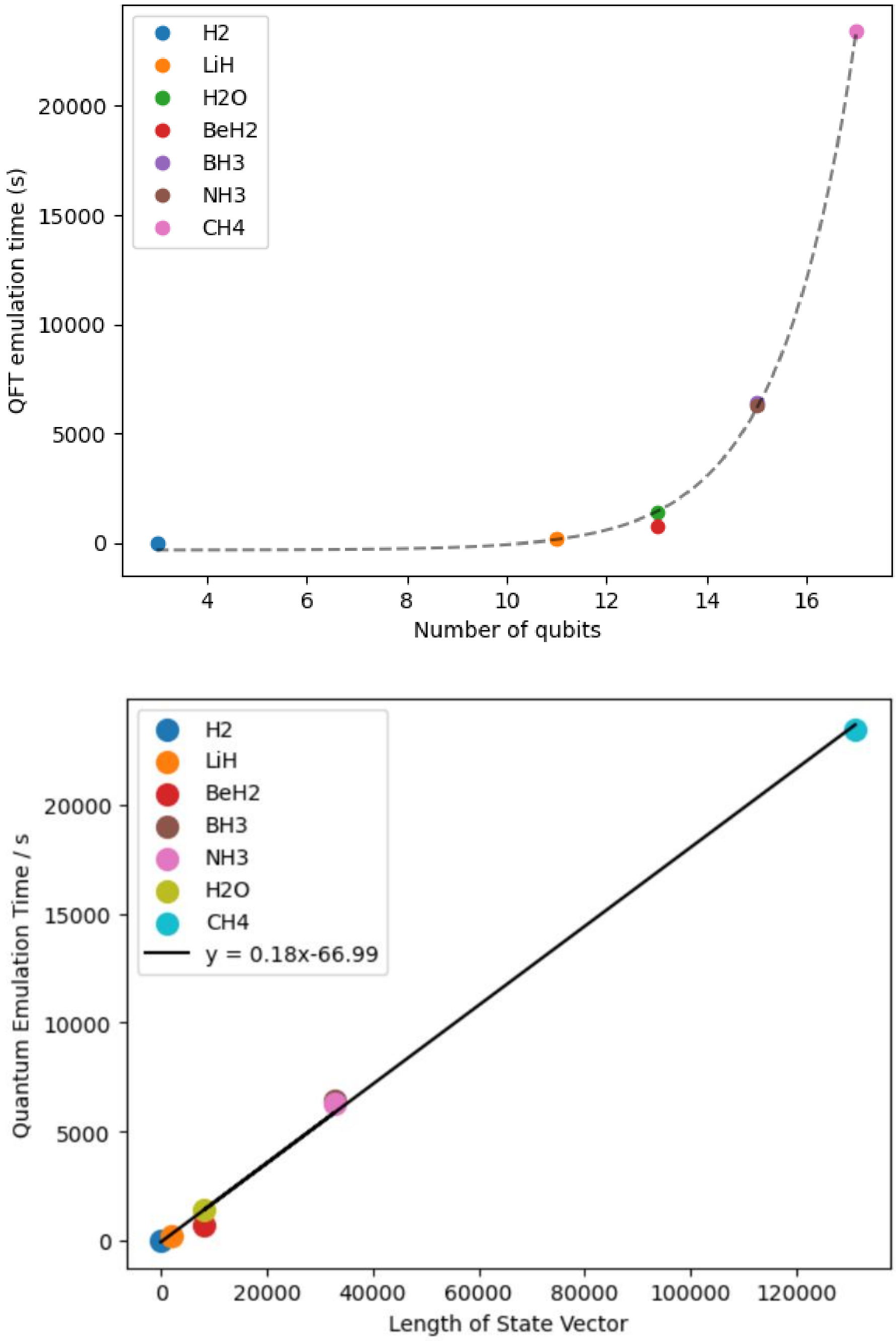

Figure S3. Computational scaling of quantum simulation on GPU (same as that described in figure S2) with respect to the number of qubits and the state vector length. The state vector for even a small molecule (i.e. CH_4) is extremely large as it scales 2^N, where N is the number of qubits required for the computation. The runtime cost of quantum emulation scales linearly with the size of the state vector and exponentially with the number of qubits.

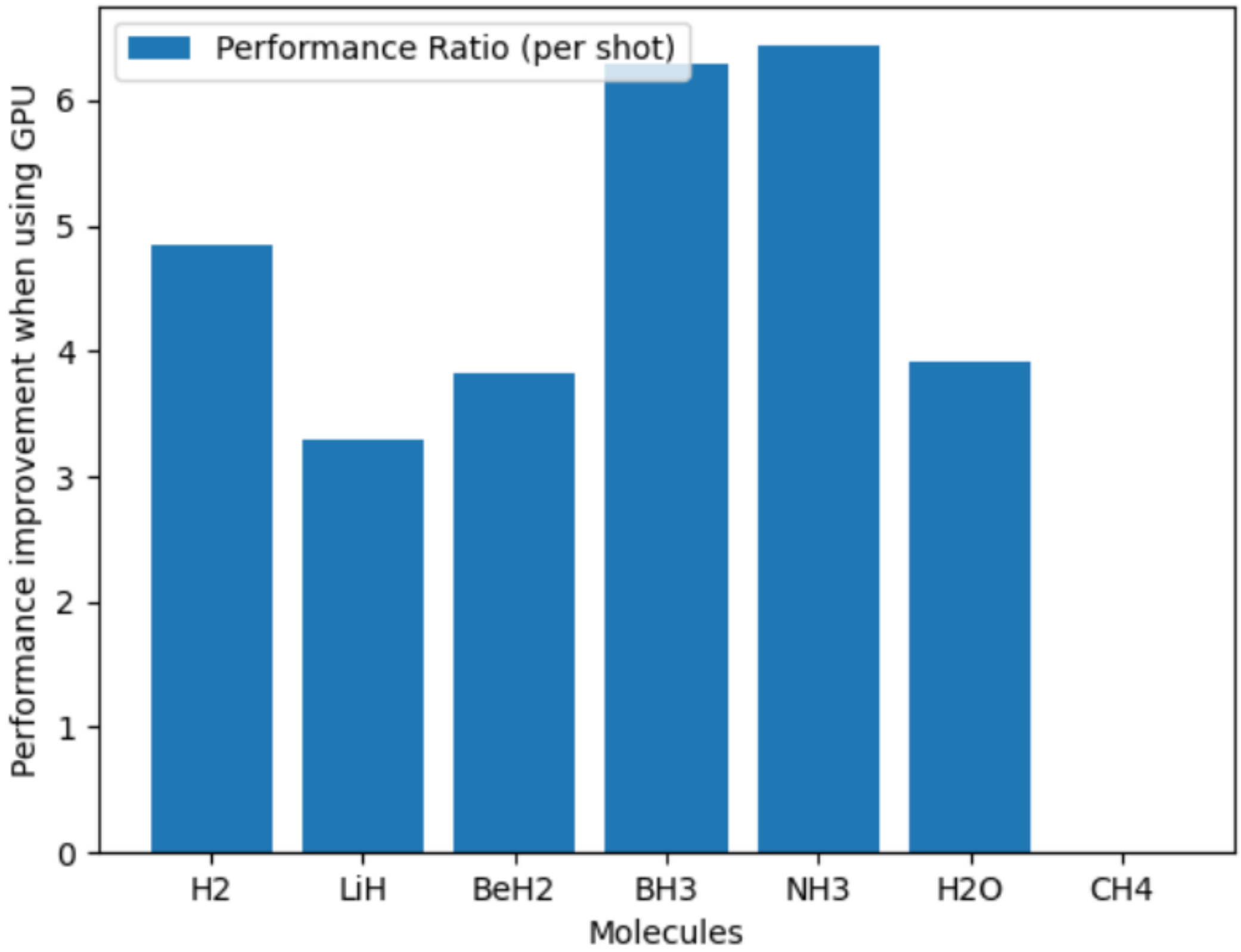

Figure S4. The relative speed-up when comparing the circuit emulation on CPU (shown in Figure S1) compared to GPU (shown in Figure S2). It is shown that for the largest systems (i.e. BH_3 and NH_3) there is an approximate performance improvement of 6x when using GPU. CH_4 data is not available due to inhibitive computational cost on CPU.

Molecule	# of qubits	Depth	Depth (2 qubit)
Paraquinone (4,3)	5	1,690,822	1,134,038
Paraquinone (4, 4)	7	6,030,462	4,167,692
Paraquinone (6,5)	9	28,853,370	20,352,150
Paraquinone (6,6)	11	128,500,408	91,788,346
Paraquinone (8,7)	13	258,000,118	185,689,970
Paraquinone (8,8)	15	908,273,520	658,367,238
Paraquinone (10,9)	17	1,364,638,368	997,691,934

Table S1. Quantum computational resource requirements of the innermost layer (QPE) with different active spaces sizes for paraquinone, where the first number is the number of electrons and the second is the number of spatial orbitals.
